# Prevalence of pernicious anemia in patients with macrocytic anemia and low serum B12 

**DOI:** 10.12669/pjms.306.5413

**Published:** 2014

**Authors:** Abdulmanea AA, Alsaeed AH, Shaik AP, AlGahtani FH

**Affiliations:** 1 Abdulmanea AA, MSc, College of Applied Medical Sciences, King Saud University, Riyadh, Saudi Arabia.; 2 Alsaeed AH, PhD, College of Applied Medical Sciences, King Saud University, Riyadh, Saudi Arabia.; 3 Shaik AP, PhD, College of Applied Medical Sciences, King Saud University, Riyadh, Saudi Arabia.; 4 Al-Gahtani FH, MD, College of Medicine, King Khalid University Hospital, Riyadh, Saudi Arabia.

**Keywords:** Pernicious anemia, Megaloblastic anemia, Macrocytic anemia, Vitamin B12

## Abstract

***Objective:*** The current research evaluated the prevalence of pernicious anemia (PA) in patients with macrocytic anemia (high MCV) and low serum B12 in Riyadh.

***Methods: ***Blood testing was done in 77 patients (males: 45.5%, females: 54.5%) with macrocytic anemia; 84 patients; (males: 23.8%, females: 76.2%) with low serum B12 and 30 healthy subjects. Complete blood count, differential count, folic acid, vitamin B12, intrinsic factor, gastric parietal cell antibodies and holotranscobalamin II were assessed.

***Results: ***A total of five subjects from 161 patients had PA; three of these patients had macrocyticanemia (3.90%) and two patients had low serum B12 (2.38%). Significant differences (p<0.05) in some hematological, immunological, biochemical parameters were found in subjects with macrocytic anemia and low serum B12 compared to controls.

***Conclusions:*** Pernicious anemia in patients with macrocytic anemia and low serum B12 was for the selected sample size can be assumed to be uncommon in Riyadh, Saudi Arabia.

## INTRODUCTION

Pernicious anemia (PA) is a chronic illness where body fails to produce normal healthy red blood corpuscles (RBCs) because of deficiency of vitamin B12 required for normal cell growth.^1^^-^^3^ The deficiency of Vitamin B12 occurs because of reduced dietary intake or impaired absorption due to lack of intrinsic factor as a result of gastric atrophy which is considered quite common in adults or as congenital hereditary autoimmune disorder.^2^ Long standing undiagnosed cases can lead to severe health problems resulting in permanent damage to vital organs such as nerves, heart and other parts of the body.^1^

Harakati reported eighteen cases of PA in Arab population between 1986 and 1994.The mean age at presentation was 51 years and the frequency of anti-intrinsic factor antibodies was very high, 89%.^1^ Harakati also studied the incidences of PA in Saudi Arabian population between 1986 and 1990, seven cases were diagnosed in Riyadh.^2^^,^^3^ A study from the United Arab Emirates described six PA patients and five of the subjects had signs or symptoms of neurologic dysfunction and nerve conduction.^4^ Al-Ajmi reported that the PA is uncommon in Arab.^5^

Looking at this discrepant data, the current research was aimed to determine the prevalence of PA in patients with macrocytic anaemia (high mean corpuscular volume [MCV]) and in patients with low serum B12 visiting the King Khalid University Hospital (KKUH) in Riyadh.

## METHODS

***Patients:*** Blood samples from a total of 77 adult patients (35 males, 42 females) having macrocytic anemia (MCV >99 fl) and 84 adult patients (20 males, 64 females) with low serum B12 (B12 <145 pmol/L) and from 30 healthy volunteers (21 males, 9 females) were collected. Evidence of megaloblastic haematopoiesis included features shown by peripheral blood smear and red cell indices–MCV>99 fL, presence of ovalomacrocytes and/or hypersegmented neutrophils. Hypersegmentation was defined as >5 five-lobed cells/100 neutrophils or any one neutrophil with >6 lobes.

Eligibility criteria included adult patients above 16 years and below 75 years of the age. Pregnant and lactating women and patients receiving any hormonal treatment, sickle cell disease, patients with a history of surgery/surgeries or exposure to chemotherapy or irradiation, patients with any evidence of systemic diseases or chronic illness, and patients consuming iron supplements within the past four months were excluded.

This study was approved by Institutional Review Board (IRB) at KKUH Council of Medical Research Ethics and signed written informed consent was obtained from all subjects. Blood specimens were investigated for complete blood cell count, folic acid, vitamin B12, intrinsic factor, parietal cell antibodies and holotranscoblamine II.

***Assessments*** **:** Hematological values were measured on full Automatic blood cell counter hematology analyser (Beckman Coulter^® ^UnicelDxH 800). Vitamin B12 and serum folic acid was measured using fully automated electrochemiluminescence immunoassay (ECLIA) reagent in Modular analytic E170 analyser. Serum Holotranscoblamine II (Active B12) was measured by fully automated AxSYM-Abbott Diagnostics, chemiluminescent microparticle immunoassay (CMIA) for quantitative determination of Holotranscoblamin in human serum on Architect System (3P24-25).^6 ^Serum IF antibodies were measured by Quanta Lite TM Intrinsic Factor ELISA (708780) INOVA using Diasorin ETI-max 3000 (Via Crescentino SNC - 13040 Saluggia (VC) Italy).^7^ **S**erum GPC antibodies were measured by indirect immunofluorescence on mouse-stomach sections (NOVA Lite® ANA Plus).^8^

***Statistical analyses:*** All analyses was performed using SPSS software (SPSS Inc., Chicago) employing simple proportion t-test, independent t-test, one away analysis of variance (ANOVA) and odds ratio. A p <0.05 was considered significant.

## RESULTS

The clinical characteristics of patients diagnosed as having PA are presented in Tables I and II. A total of 5 patients (3 males and 2 females, two subjects with low B12 and three subjects with macrocytic anemia) were positive for IF antibody (46.5-99.9 units). Of these, two subjects had low B12 and three patients had high MCV levels. The active B12 levels ranged from 6.9-130 while folate levels ranged from 10.0-35.5. The B12 levels varied widely and ranged from 105–950.

The mean values of MCV in the control group (Males: 86.09±3.2 and Females: 87.83±4.5) and serum B12 levels (Males: 327.52±137.0 and females: 361.44±35.5) were comparable with no statistically significant difference (p=0.09) in both genders. There was a statistically significant difference between males and females in patients with macrocytic anaemia and low B12 values (p< 0.000) (Table-III). Among 77 patients with macrocytic anaemia patients 18.3% had low serum B12 level (<145pmol/L), 16.9% had high serum B12 (>637 pmol/L) and 64.8% had normal serum B12 levels (145-637pmol/L). Among 84 patients with low B12 values, 23.8% had low MCV (< 80 fL), 3.6% had high MCV (>99fL) and 72.6% had normal MCV (80-99 fL).

The comparison of mean values of haematological, immunological and biochemical parameters of patients with macrocyticanemia, patients with low serum B12 and the control group are shown in Tables IV and V. A significant increase (p<0.000) in MCV, RDW, serum B12, folate and holotranscoblamin (active B12) values were observed among patients with macrocytic anemia, low serum B12 and PA compared to controls.

## DISCUSSION

PA is macrocytic anemia due to vitamin B12 deficiency as a result of deficiency of intrinsic factor because of the atrophy of gastric mucosa, and thus, the loss of parietal cells.^9^ Varying incidence rates of PA have been reported.^10^^,^^11^ The present study evaluated the incidence of PA in patients with macrocytic anemia and in patients with low serum B12.

Demographic features such as age and sex did not affect the study results. In concordance with previous studies,^12^^-^^14^ the mean MCV and mean serum B12 were 79-90 fL and327-361 respectively. There was no significant difference in haematological and biochemical parameters between males and females in patients with macrocytic anemia. The mean values of MCV, RBC, haematocrit, platelets, RDW and folate levels were found to increase significantly when compared to controls **(p<0.05).** Since macrocytosis is the earliest finding seen in patients with folate or vitamin B12 deficiency, in most cases, the classic expression of vitamin B12 deficiency is a macrocytic anaemia with accompanying elevated MCV.^15^ Similar to the study by Savage et al, there was statistically significant increase in MCV levels in patients with macrocytic anaemia. Holotranscobalamin test was used as an alternative for early diagnosis of vitamin B12 deficiency.^16^ Of 77 patients, macrocytosis with MCV >99 fL was present in 76 (98.7%) patients. There were elevated levels of folic acid in patients with macrocytic anaemia which was statistically significant. Similar finding was reported by Ashraf et al.^17^ Analogous observations of Snow indicate that the increased concentrations of folate within RBCs can falsely elevate serum folate levels through haemolysis.^18^ Vitamin B12 levels may be reported as normal or elevated in myeloproliferative disorders, liver disease, congenital transcobalamin II deficiency, intestinal bacterial overgrowth and antecedent administration of vitamin B12.^18^

**Table-I:** Clinical characteristics of patients diagnosed by screening as having pernicious anemia

**Variables**	**Patient 1**	**Patient 2**	**Patient 3**	**Patient 4**	**Patient 5**
MCV (fl)	80	93.8	103	102	106
MCH (pg/cell)	25.4	31.6	33.0	32.4	35.9
RDW (%)	18.0	15.2	18.4	25.9	28.2
HGB (g/dl)	12.6	15.5	7.9	7.4	8.7
Serum B12 (pmol/l)	121	105	520	345	950
Serum Folate (nmol/l)	22.5	35.5	21.4	10.0	23.7
Active-B12 (pmol/l)	25.0	6.9	85.3	76.6	130
PCA	Positive	Positive	Positive	Positive	Positive
IFA	Positive (52.8 Unit)	Positive (49.4 Unit)	Positive (46.5 Unit)	Positive (99.9 Unit)	Positive (98.1 Unit)
Sub-group	Low B12 patient	Low B12 patient	Macrocytic patient (High MCV)	Macrocytic patient (High MCV)	Macrocytic patient (High MCV)

**MCV**=Mean Corpuscular Volume, **MCH**=Mean Cell Hemoglobin,

**RDW**=Red Blood Cell Distribution Width, **HBG**=Hemoglobin,

**PCA**=Parietal Cell Antibodies,** IFA**=Intrinsic Factor Antibodies.

**Table-II:** The clinical characteristics (mean and standard division) of the two groups of patients with pernicious anemia

***Variables***	**Pernicious anemia with macroctyticanemia** **(Mean ± SD)**	**Pernicious anemia with low serum B12** **(Mean ±SD)**	**P- value** **t-test**
MCV (fl)	103.7±2.08	86.5±9.76	0.019*
MCH (pg/cell)	33.8±1.87	28.5±4.38	0.998
RDW (%)	24.2±5.12	16.6±1.98	0.425
HGB (g/dl)	8±0.66	14.1±2.05	0.996
Serum B12 (pmol/l)	605±311.33	113±11.31	0.001*
Serum Folate (nmol/l)	18.4±7.34	29±9.19	0.835
Active-B12 (pmol/l)	97.3±28.65	15.9±12.80	0.242

**MCV**=Mean Corpuscular Volume, **MCH**=Mean Cell Hemoglobin,

**RDW**=Red Blood Cell Distribution Width, **HBG**=Hemoglobin

**Table-III:** The mean MCV and B12 values of subjects in the control group and patients with macrocytic anemia and low vitamin B12 according to gender

***Variables***	**Control group** **Mean± SD**		**Macrocytic patients ** **Mean± SD**		**t- test**	**Low B12 patients** **Mean± SD**		**t-test**
Gender	***Male***	***Female***	***Male***	***Female***	***p-value***	***Male***	***Female***	***p- value***
MCV (fl)	86.09±3.32	87.83±4.59	107.47±8.1	105.17±5.4	0.000*	86.08±7.50	85.064±6.43	0.879
Serum B12 (pmol/l)	327.52±137	361.44±35.5	422.3±257.8	375.6±241	0.670	97.67±18.92	99.58±23.65	0.000*

**MCV**=Mean Corpuscular Volume.

**Table-IV:** The comparison of haematological, immunological and biochemical parameters of patients with macrocytic anemia and low vitamin B12compared to the control group

***Variables***	**Control Mean±SD**	**Macrocytic Patients Mean±SD**	**t- test ** **(p-value)**	**Low B12 Patients Mean±SD**	**t- test** **(p-value)**
WBC (x10^9^/l)	6.19±1.5	8.32±5.79	0.062	8.5±2.73	0.282
RBC (x10^9^/l)	5.34±0.38	2.96±0.74	0.000*	4.40±0.65	0.000*
HGB (g/dl)	15.23±0.66	11.63±12.88	0.14	12.11±2.13	0.21
HCT (%)	41.41±3.42	30.99±7.41	0.000*	36.95±2.14	0.004*
MCV (fl)	86.61±0.75	106.36±6.83	0.000*	85.32±6.67	0.629
MCH (pg/cell)	29.27±0.79	34.90±3.14	0.432	28.00±3.15	0.899
MCHC (gm/l)	336.86±6.66	329.08±21.34	0.136	328.58±13.09	0.354
RDW (%)	12.98±0.63	20.41 ±6.74	0.000*	15.85±3.30	0.022*
PLT (x10^9^/l)	316.33±72.82	224.25±135.45	0.000*	262.96±86.38	0.068
Serum B12 (pmol/l)	337.70±135.15	396.79±248.13	0.262	99.12±22.52	0.000*
Serum Folate (nmol/l)	15.11±4.09	26.4±11.96	0.000*	24.99±12.06	0.000*
Active-B12 (pmol/l)	70.36±30.28	70.13±37.55	0.999	33.96±28.89	0.000*

**WBC=**White blood cells, **RBC**=Red blood cells, **HBG**=Hemoglobin, **HCT**=Hematocrit,

**MCV**=Mean Corpuscular Volume, **MCH**=Mean Cell Hemoglobin,

**MCHC**=Mean Cell Hemoglobin Concentration,** RDW**=Red Blood Cell Distribution Width, **PLT=**Platelets.

**Table-V:** Comparison of haematological, immunological and biochemical parameters of the control and patients with pernicious anemia (PA)+macrocytic anemia and in patients with PA+low vitamin B12

**Variables**	**Control (Mean±SD)**	**PA in macrocytic** ** patients (Mean±SD)**	**T- test (p-value)**	**PA in low B12 patients ** **(Mean ± SD)**	**T- test (p-value)**
WBC (x10^9^/l)	6.19±1.5	8.300 ±3.48	0.919	8.45±0.64	0.946
RBC (x10^9^/l)	5.34±0.38	2.99±0.058	0.000*	5.05±0.21	0.973
HGB (g/dl)	15.23±0.66	8.00±0.66	0.608	14.05±2.05	1.000
HCT (%)	41.41±3.42	24.27±1.40	0.000*	42.45±5.16	0.999
MCV (fl)	86.61±0.75	103.7±2.89	0.000*	86.5±13.08	0.992
MCH (pg/cell)	29.27±0.79	33.77±1.87	0.996	28.5±5.09	1.000
MCHC (gm/l)	336.86±6.66	329.33±9.07	0.991	330.50±9.19	0.998
RDW (%)	12.98±0.63	24.17±5.13	0.002*	16.60±1.98	0.031*
PLT (x10^9^/l)	316.33±72.82	90.33±26.39	0.005*	263.56±37.48	0.069
Serum B12 (pmol/l)	337.70±135.15	605.00±430.67	0.005*	112.50±10.61	0.000*
Serum Folate (nmol/l)	15.11±4.09	18.37±7.34	0.989	29.00±9.19	0.000*
Active-B12 (pmol/l)	70.36±30.29	97.300±28.65	0.775	15.90±26.87	0.000*

**WBC=**White blood cells, **RBC**=Red blood cells, **HBG**=Hemoglobin, **HCT**=Hematocrit,

**MCV**=Mean Corpuscular Volume, **MCH**=Mean Cell Hemoglobin,

**MCHC**=Mean Cell Hemoglobin Concentration,** RDW**=Red Blood Cell Distribution Width, **PLT=**Platelets.

In this present study, IF antibody and GPC antibody test was used to confirm diagnosis of PA.^19^ The serum antibody to IF and GPC was detected in 3 (3.9%) and 5 (6.5%) of 77 patients with macrocytic patients respectively. The mean of RBC, MCV, haematocrit, platelets, RDW and serum B12 were found to be significantly high compared to controls (p<0.000). Both serum antibody to IF and GPC were detected in 3 (3.9%) of macrocytic patients and were therefore diagnosed as having latent PA. In normal subjects, there seems to be an age-related increase in the incidence of antibodies to GPC from 2 to 8%. Also there was a general increase in the number of persons with atrophic gastritis with the progress of age.^20^ Similar to the study reported by Van Rossum et al.^21^, we have reported that there was an elevation of B12 level (950 pmol/L) for one patient with PA and macrocytic anemia.

Corroborating the findings of Carmel^22^, high MCV and anemia (low Hb) were observed. The RDW value was higher in all patients with PA. Calvo Romero et al., also reported higher RDW in PA.^23^ All patients with PA were positive for parietal cell and intrinsic factor antibodies. A similar study by Kyle reported that 90% of patients with PA tested positive for one or both of parietal cell and intrinsic factor antibodies.^24^

Results of 84 low serum B12 patients showed significant differences in the serum B12, folate, active-B12, RBC, HCT, and RDW values of patients with low serum B12 (P< 0.000), and no significant differences were observed for the remaining tests. These results are congruent with studies conducted by Jain et al.^25^ An elevation of folic acid levels in patients with low B12 levels was observed. Comparable results were reported by Chanarin and Metz.^12^

Determination of holotranscobalamin concentrations may be used as a complementary diagnostic strategy to avoid the development of pathological conditions (macrocytic anemia or neurological disease) before symptoms emerge, and should also be used for large scale screening of subjects at latent risk of B12 deficiency. All patients with low serum B12 were tested for serum antibodies to IF and GPC. Serum antibody to IF and GPC was detected in 2 (2.4%) and 11 (13.1%) among 84 low serum B12 patients examined. Antibodies for IF and GPC were detected in 2 patients (2.4%) one male, one female and diagnosed as having latent PA. GPC antibodies were seen in about 13.1% of patients with low serum B12 levels but not all patients were diagnosed as having PA. In summary, PA in macrocytic anemia patients and in subjects with low serum B12 patients was uncommon in Riyadh (Saudi Arabia), although, these results can be hypothesized for larger population size, further studies are needed especially in additional provinces of Saudi Arabia for firm conclusions.

## Notes

***Source of funding:*** Deanship of Scientific Research, King Saud University, Riyadh through the research group project No RGP-VPP-259.
